# Online information seeking and attitudes towards COVID-19 vaccination in Germany

**DOI:** 10.1093/eurpub/ckac131.179

**Published:** 2022-10-25

**Authors:** L Schmidt, B Babitsch

**Affiliations:** School of Human Sciences, Osnabrück University, Osnabrück, Germany

## Abstract

**Background:**

A large proportion of the European population seeks information about the COVID-19 vaccination on the internet. The population seems to split into those with positive stance on the vaccination and those with negative stance, but there are still individuals who do not have a final position yet. By now, there is a lack of understanding about the online information seeking behavior in these three groups. The current analysis sheds light on differences in internet usage and requested qualities of online information regarding the COVID-19 vaccination.

**Methods:**

An online survey with N = 1,000 people (18-74 years) living in Germany was conducted between November 26 and December 8, 2021. The questionnaire included closed questions about frequency, information channels, formats and reasons of online information seeking, as well as one open question about requested qualities of online content. We conducted bivariate analysis for differences in information seeking behavior and content analysis for the requests.

**Results:**

Information seeking behavior differed significantly by attitude towards the vaccination regarding frequency, almost all types of formats, three of ten listed information channels, and three of six reasons for online information seeking. Undecided participants and those who support COVID-19 vaccination used the internet more often than participants who are against the vaccination. Individuals supporting vaccination preferred reading text contributions (e.g. online articles). Informative videos were more often consumed by those who are undecided or against vaccination. Those who have not decided yet preferred online resources providing full information about side-effects and showing reliable facts by credible sources.

**Conclusions:**

Our findings support an online vaccination communication that is tailored to target groups with different attitudes towards the vaccination. Overall, online campaigns should focus on transparent, reliable and complete information.

**Key messages:**

• Online information seeking behavior regarding COVID-19 vaccination varies between individuals with different attitudes towards the vaccination.

• Online information about the COVID-19 vaccination should focus on transparency and reliable information.

